# Metastatic cancer detection and management with artificial intelligence and augmented reality (Review)

**DOI:** 10.3892/mi.2026.297

**Published:** 2026-01-14

**Authors:** Hanisha Reddy Kukunoor, Adithya Andanappa, Kaushalendra Mani Tripathi, Iram Fatima, Ozoemena Z. Akah, Ansari Maha Faisal, Fawad Talat, Harsh Bhatia, Arlette Villalobos, Prachi Dawer, Yusra Qamar

**Affiliations:** 1 Department of Internal Medicine, Maheshwara Medical College and Hospital, Hyderabad, Telangana 502307, India; 2 Department of Internal Medicine, Mysore Medical College and Research Institute, Mysore, Karnataka 570001, India; 3 Department of Internal Medicine, White River Health Medical Center, Batesville, AR 72501, USA; 4 Department of Internal Medicine, Holy Name Medical Center, Teaneck, NJ 07666, USA; 5 Department of Internal Medicine, HCA Mountain View Hospital, Las Vegas, NV 89128, USA; 6 Surat Municipal Institute of Medical Education and Research (SMIMER), Umarwada, Surat, Gujarat 395010, India; 7 Department of Internal Medicine, Ponce Health Sciences University School of Medicine, Ponce 00716, Puerto Rico; 8 Department of Internal Medicine, University College of Medical Sciences (UCMS), New Delhi 110095, India; 9 Department of Internal Medicine, UHS Wilson Medical Center, Johnson City, NY 13790, USA; 10 Department of Internal Medicine, Danylo Halytsky L'viv National Medical University, Lviv 79010, Ukraine; 11 Department of Obstetrics and Gynecology, King George's Medical University (KGMU), Lucknow, Uttar Pradesh 226003, India

**Keywords:** metastatic cancer, artificial intelligence, augmented reality, algorithmic bias, metastatic diagnoses

## Abstract

Metastatic cancer remains a significant global health challenge, contributing to the majority of cancer-related mortality due to late detection, therapeutic resistance and the complexity of disseminated disease. Recent advances in artificial intelligence (AI) and augmented reality (AR) are transforming the landscape of metastatic cancer detection and management. AI-driven tools, including radiomics, deep learning models, and predictive analytics, enhance early identification of metastatic lesions, improve diagnostic accuracy, and support personalized treatment strategies by integrating multimodal clinical, imaging and molecular data. At the same time, AR technologies are increasingly applied in image-guided surgery, real-time tumor visualization and patient education, enabling more precise interventions and improved clinical decision-making. The combined use of AI and AR fosters multidisciplinary collaboration, facilitates comprehensive treatment planning, and may ultimately improve patient outcomes. However, despite these advancements, several challenges limit widespread implementation, including algorithmic bias, variability in data quality, concerns regarding patient privacy, and regulatory and ethical constraints. Furthermore, integration into clinical workflows requires robust validation, clinician training, and standardized guidelines. Future efforts are required to focus on developing transparent, generalizable AI models, strengthening data-security frameworks, and enhancing AR usability to ensure equitable, safe, and effective incorporation of these emerging technologies into metastatic cancer care.

## 1. Introduction

### Background on metastatic cancers. Definition of metastatic cancer

The final and most lethal stage of cancer is metastasis, which is the growth of cancer cells in organs other than the one in which they first appeared (1). Metastatic illness, rather than primary tumors, is the cause of mortality for the vast majority of patients with cancer (2). Over time, cancer cells develop the ability to penetrate deeper tissues through the mucosa; spread via the blood, lymphatics, or direct infiltration of nearby structures; seed distant organs; and finally resume proliferation in distant locations to colonize these organs (3,4). The majority of cancer cells that exit a tumor die from different stressors rather than colonizing distant organs, even though cancer cell dispersal can begin early in tumor growth. Cancer cells must navigate a sequence of events known as the ‘metastatic cascade’ to spread, and each stage calls for a distinct set of functions. Even a the response of a patient to systemic treatment can fluctuate significantly depending on whether their cancer is primary or metastatic (5). As metastatic cancers have developed a resistance to current treatments, clinically noticeable metastasis is still generally incurable with rare exceptions (6).

*Prevalence and impact on global health*. On the other hand, the distant spread of cancer identified at the time of initial diagnosis prior to the initiation of any antitumor medication is referred to as metastatic cancer. The incidence of MC is frequently linked to aggressive tumor biology, late initial tumor detection, ineffective screening methods and restricted access to suitable diagnostic tests (7,8). Even though MC may be treated, the prognosis is often worse and it is often incurable. Particularly in low-middle-income countries, where many innovative treatments are either unavailable or too expensive, MC poses a serious therapeutic issue (9).

At one of the main radiation facilities in the West African subregion, unique cross-sectional research was carried out to ascertain the prevalence, incidence and clinicopathological features of patients with metastatic cancer (10). Patients with metastatic cancer who received treatment at the study location for ~2 years were included in the research. To determine the prevalence of metastatic cancer, all patients aged ≥15 years who were verified to be cancer-treatment naïve and had been diagnosed with metastatic cancer of any primary site at the time of presentation were identified using whole population sampling. The rates of the incidence and prevalence of metastatic cancer were 5 and 15%, respectively. Overall, a sizable portion reported a history of alcohol and tobacco use. Additionally, 25% received a diagnosis of both diabetes mellitus and hypertensive heart disease at the same time. Overall, a positive family history of cancer was present in 10% of the patients. For the majority of patients, *de novo* metastases were linked to various underlying cancer locations. Disparities in healthcare access, use and the quality of care obtained by cancer patients may be closely related to the difference in the prevalence of metastatic cancer between high- and low-income countries (10-12). Early cancer identification and a lower chance of metastatic diagnoses are made possible by the often better developed healthcare systems of high-income nations, extensive cancer screening programs and increased public health awareness (11,12). On the other hand, low-income nations, such as Ghana frequently have challenges with restricted access to healthcare, fewer screening programs, and a lack of knowledge about cancer, which results in a higher number of advanced-stage cancer diagnoses (13).

*Challenges in detection and management*. In addition to addressing the dynamic plasticity of the cancer cells as they move through the metastatic cascade, the effective therapeutic targeting of metastatic cancer must also address the strategies employed by both dormant and growing metastases to coopt and corrupt their niches and elude immune surveillance (14,15).

The early identification of metastatic cancer is challenging as it frequently spreads before symptoms appear. Imaging scans may not reveal small metastatic lesions. Given the heterogeneity of metastases, a single biopsy is insufficient for a complete diagnosis, since various metastatic locations may contain distinct genetic mutations (16). Since the brain is usually the final location of metastasis relapses, it is a case for organ-specific therapy. Cancers that spread to these clinically difficult areas are expected to have better outcomes if the metastatic mechanisms in these particular microenvironments are better understood and targeted (17).

Given that macro metastatic or clinical stage IV illness is still primarily incurable, it is clear that metastasis and therapeutic resistance are closely related (18). Although sensitivity and specificity remain as issues, liquid biopsies can be useful, particularly for identifying very early-stage metastases, particularly in situations of circulating tumor cells and circulating tumor DNA detection (19). Despite experimental and clinical evidence to indicate that metastasis begins early, medications that target mediators of metastatic dissemination, such as matrix metalloproteinase inhibitors, have not yet exhibited promise in clinical trials (20).

As the field of cancer nanomedicine has advanced significantly, it is evident that various opportunities and issues lie ahead. First and foremost, it is evident that rigorous patient selection is necessary to determine which patients are most likely to benefit from a particular nanotherapy due to the intricacy and heterogeneity of tumors. This is comparable to the targeted treatments that have been authorized or are being developed for use in patient populations that are identified by particular biomarkers (21). The enhanced permeability and retention effect, which is usually believed to be the result of leaky tumor vasculature, is how the majority of therapeutic nanoparticles (NPs) for the treatment of solid tumors aggregate in the tumor when administered systemically (22).

### Significance of early detection and accurate management. Importance of early diagnosis in improving outcomes

Even in the event that metastatic cancer has already spread beyond its original location, early detection is essential for improving patient outcomes. Early metastatic detection enables prompt therapy measures, which can prolong survival and reduce the course of the illness (23).

Oncologists can investigate a greater variety of therapeutic options when metastases are detected early, such as systemic medicines (chemotherapy, immunotherapy and hormone therapy) and local treatments such as surgery or radiation for isolated metastases (24). This may be useful since, with a few exceptions, clinically visible metastasis continues to be mostly incurable as metastatic cancers have developed acquired resistance to current treatments (25).

*Limitations of traditional detection and treatment methods*. Microscopic metastatic lesions may be undetected by standard imaging methods (CT, MRI and PET scans), delaying detection. The illness may have progressed by the time metastases become apparent. Late-stage detections may result from the silent metastases of some malignancies (such as pancreatic or ovarian) that remain undetected for a long period of time (26,27). Particularly for deep-seated organs such as the brain and lungs, tissue biopsies are intrusive, dangerous and unpleasant. Tumor heterogeneity may be missed by a single biopsy, resulting in an incomplete diagnosis. As there are no real-time monitoring in between planned scans, tumor development or response to therapy may go undetected. Treatment choices may be delayed if imaging visits and biopsy findings are not obtained on time (23).

### Role of technology in cancer care of emerging technologies in oncology

The particularly alluring qualities of nanotechnology for medication administration, detection and imaging, the creation of synthetic vaccines, and small medical devices are primarily responsible for its association with cancer. The clinical research of nanotherapies that combine some of these characteristics is very promising (28). A number of therapeutic NP platforms, including polymeric micelles, albumin NPs and liposomes, have been authorized for the treatment of cancer (28).

As the tumor microenvironment influences a number of facets of tissue homeostasis, the majority of aggressive cancers frequently recur after receiving standard therapies, including chemotherapy, radiation therapy, or, where practical, surgical resection (29).

Therefore, 3D models with improved immuno-modulatory, proliferation and activation capabilities include melanoma-derived spheroids. Notable advancements have been achieved in identifying novel therapeutic targets, uncovering mutant genes, and identifying precise diagnostic biomarkers through the use of single-cell sequencing technologies in the study of various types of cancers (30).

Additionally, oncologists need to improve their ability to communicate the demands of the cancer care system to governmental organizations and public decision-makers. Institutions should pay attention to some of the consequences of innovation (31).

### Novelty and distinction from previous reviews

The applications of artificial intelligence (AI) and augmented reality (AR) in metastatic cancer management, the majority have primarily focused on either diagnostic imaging or surgical guidance in isolation. The present review distinguishes itself by providing a comprehensive integration of AI and AR across the entire metastatic cancer care continuum, from early detection and predictive analytics, to intraoperative visualization and patient education. Moreover, the present review emphasizes technical implementation details, such as data preprocessing, AR spatial registration and latency optimization. Additionally, it includes real-life case analyses (e.g., AI-assisted colorectal histopathology, AR-guided hepatic metastasectomy) and discusses ethical challenges with practical mitigation strategies, such as bias correction frameworks and data governance standards. This multifaceted and implementation-oriented perspective provides unique translational insight, bridging the gap between experimental research and real-world clinical application.

### Introduction to AI and AR

AI and AR are rapidly evolving technologies that have found extensive applications across various domains, particularly in healthcare, education and industry. In recent years, orthopedics and other surgical specialties have pioneered their integration, marking several ‘first steps’ in the field of precision medicine (32).

AR focuses on seamlessly merging digital and virtual information into a single visual representation; however, it lacks the capability for complex scene interpretation on its own (33). Therefore, AR systems are often integrated with AI, particularly computer vision, to enable 3D model registration, object segmentation and pose estimation.

AI data preprocessing plays a critical role in ensuring model accuracy and robustness. Common steps include image normalization, noise filtering, data augmentation (e.g., rotation, scaling and contrast adjustment), and dimensionality reduction using principal component analysis or autoencoders to prevent overfitting. In clinical imaging workflows, preprocessing typically involves DICOM-to-NIfTI conversion, segmentation mask refinement and voxel intensity normalization prior to deep learning model training (32-34).

For AR integration, precise spatial registration between virtual 3D models and patient anatomy is achieved through real-time tracking using fiducial markers or sensor-based navigation. System calibration minimizes overlay error, while graphics processing unit-accelerated rendering and predictive motion correction algorithms reduce latency to maintain visual stability during intraoperative use (33,34).

The fusion of AI-based image analysis with AR visualization enhances intraoperative precision, particularly in tumor localization, margin assessment and resection planning. These integrated approaches have been effectively demonstrated in colorectal and hepatic oncology applications, highlighting their growing clinical relevance in precision surgery (32,33).

### AI in cancer detection and management

AI is a branch of science and engineering that focuses on the creation of artifacts that exhibit intelligent behavior and on the computational understanding of what is often referred to as intelligent behavior. In medicine, this entails data analysis, image interpretation, pattern recognition and decision support. AI tools are capable of analyzing complex medical data. Their potential to exploit key relationships within a data collection can be used in the diagnosis, treatment and prediction of outcomes in various clinical contexts (34). They are intended to assist healthcare professionals with tasks that require the manipulation of data and expertise as part of their daily responsibilities. Artificial neural networks, fuzzy expert systems, evolutionary computation and hybrid intelligent systems are examples of such systems (34).

Cancer is a substantial contributor to worldwide mortality, accounting for 9.3 million deaths per year, driving the investigation of AI applications in numerous oncology fields to reduce this burden (35). AI, which uses mathematical algorithms to simulate human cognitive capacities, first appeared in medicine as rule-based expert systems that provided decision support based on established criteria. Over time, advances in computer power and the availability of massive data sets drove the evolution of these primitive systems into sophisticated machine learning (ML) and deep learning (DL) techniques. These cutting-edge technologies can now detect patterns and abnormalities that indicate early cancer stages, allowing for earlier medical treatments (36).

Notable milestones in this evolution include the initial application of AI for radiographic image interpretation and recent developments in genomic data analysis, which have been critical in personalizing cancer treatment by analyzing genetic markers and variations to predict predispositions and treatment results. Furthermore, the ability of AI extends to biometric data analysis, in which wearable sensors continuously monitor vital signs for variations that may indicate future health risks (36,37). This booming subject is indicated by a steady increase in research productivity, with 1,161 authors publishing 214 articles in 133 journals throughout 1988 and 2021, along with key contributions from significant research hubs that include the USA and Korea (38). As the scientific community continues to expand its collaborative networks, current biomedical research remains focused on safely and ethically translating AI technology into clinical practice, addressing the complex, multifaceted nature of cancer, a disorder characterized by thousands of genetic and epigenetic variations (38).

AI algorithms have considerably improved disease diagnosis by efficiently analyzing data from a variety of radiological imaging modalities. In radiology, these technologies are extremely precise in detecting abnormalities including tumors, fractures and early indicators of disease (36). For example, in digital breast tomosynthesis (DBT), AI models aid in breast cancer detection by lowering reading rates and increasing both incremental and overall cancer detection rates, all while streamlining the clinical workflow by reducing the number of images requiring direct review by physicians (39). Deep learning algorithms have made it easier to analyses X-rays and low-dose CT scans for lung cancer screening. Although low-dose CT scans are intrinsically more accurate than X-rays, AI integration improves their diagnostic ability. Convolutional neural networks (CNNs) have been designed to analyze chest X-ray images in conjunction with clinical parameters, such as age, sex and smoking history in order to identify individuals who are at a high risk of developing lung cancer. These AI solutions not only increase diagnostic accuracy, but may also enable early intervention, resulting in improved patient outcomes in radiology. Similar such AI models have been trained for cervical cancer, skin cancer, oral cancer, esophageal squamous cell carcinoma, adenocarcinoma of the esophagogastric junction, adenomas of the colorectum and polyps (39).

The digitization of tissue slides and rapid advances in AI, particularly deep learning, have transformed computational pathology by automating clinical diagnosis, forecasting patient prognosis and medication response, and identifying novel morphological biomarkers (40). This paradigm shift is reshaping pathology services to meet the needs of precision medicine. Digital pathology is increasingly used in cancer diagnosis, with automated whole slide imaging scanners producing high-resolution images of complete glass slides that incorporate morphological, clinical, and molecular data into pathology reports (41). ML techniques, particularly convolutional neural networks, are widely employed for a variety of applications, ranging from simple cell object recognition to complicated visual pattern recognition for disease diagnosis, outcome prediction and therapeutic guidance. These techniques exploit image patches to train models that accomplish quantitative histomorphometric evaluations, identifying characteristics such as nuclear orientation, texture, and architecture from typical hematoxylin and eosin slides (41). By automating time-consuming processes, AI raises diagnostic accuracy and efficiency, improves inter-pathologist concordance on subjective assessments (including cytonuclear pleomorphism and mitotic figure counts), and even detects subtle patterns in the tumor microenvironment that humans cannot recognize. In addition, AI-powered content-based image retrieval systems can swiftly scan large histopathology datasets for comparable instances, assisting in the identification of rare and difficult illnesses (42).

Predictive analytics has been transformed by the application of AI in oncology, which has improved therapy response prediction, prognosis accuracy and individualized patient care (43,44). While AI-driven ML and DL models examine large datasets from wearable technology, electronic health records and multi-omics data to produce personalized forecasts, traditional statistical models frequently fail to capture the complexity of cancer progression, these algorithms let physicians more accurately estimate survival, predict tumor recurrence, forecast metastasis and assess cancer risk (45).

AI greatly enhances the early detection and stratification of cancer risk. For example, compared to standard screening guidelines, an artificial neural network model for colorectal cancer risk stratification has shown superior accuracy, and a similar model for pancreatic cancer yielded an area under the receiver operating characteristic curve of 85% (39). These models enable more rapid detection and improved treatment outcomes, rendering them particularly useful for cancers without defined screening regimens. Beyond early detection, AI predicts the response of each patient to treatment, optimizing therapeutic approaches (39,46,47). To improve treatment planning, AI-driven decision support systems combine real-world clinical data with proteomic, transcriptomic and genomic data (39,47). This method guarantees that patients receive the optimal treatments possible and enables genuinely personalized care. AI also improves wearable equipment for remote patient monitoring, allowing for prompt medical interventions and early detection of possible health declines (46). AI is influencing the future of oncology by facilitating customized treatment plans, increasing diagnostic accuracy and optimizing clinical operations. The contribution of AI to cancer treatment will only grow as computing power increases, enabling a more accurate and effective method of patient care.

Torrente *et al* (46) trained an AI model, Cancer Long Survivor Artificial Intelligence Follow-Up (CLARIFY), using clinical data from 5,275 patients diagnosed with non-small cell lung cancer, breast cancer and non-Hodgkin lymphoma at Hospital Universitario Puerta de Hierro-Majadahonda. Data from 5,275 patients with cancer were analyzed using an AI tool that included wearable device data, clinical data and questionnaire data. Patients with low and high-risk profiles were identified using a prognostic model, survival probabilities were computed, and descriptive analyses were conducted to examine the features of the patients. The data integration of the CLARIFY platform provides physicians with a new tool for accessing and analyzing data in real-time. This tool enables survival analyses based on certain pathology and treatment aspects, cohort descriptive analysis based on chosen parameters, and cancer patient profiling. Additionally, it enables clinicians to compare the behavior and results of their patients with those of larger series or clinical trials. It can also help clinicians in their day-to-day clinical practice by determining the risk of relapse in a particular patient profile, stratifying a selected patient or group of patients, and analyzing their behavior in terms of specific disease aspects that cannot be analyzed in any other way, such as the effectiveness of follow-up tests or the response to a particular drug (46).

The application of AI in healthcare, particularly in precision medicine, is severely hampered however, by issues with data quality, integration and bias (45,46). AI-driven prediction models require big, high-quality datasets; regrettably, present biological data is frequently scattered among multiple incompatible databases, rendering integration problematic (48). Inconsistent data quality challenges the creation of accurate predictive models, since incomplete or biased datasets may lead to incorrect conclusions. Managing the complexity of biomedical big data, which is characterized by massive volume, high velocity and extensive variation, remains a significant challenge (46). Furthermore, ethical issues are raised about patient privacy and data security; AI systems require access to sensitive personal health data, which raises the possibility of breaches, misuse and ethical quandaries connected to consent and confidentiality (45,46,48) Ensuring suitable precautions while maintaining the analytical power of AI is critical.

Another major concern is algorithmic bias, which occurs when AI models are trained using past healthcare data that may reflect existing discrepancies in treatment and patient demographics (48,49). If not addressed, such biases can perpetuate disparities in healthcare results, limiting the ability of AI to provide truly fair and personalized therapy. To overcome these limitations, standardized, high-quality datasets, improved data-sharing mechanisms and ethical AI governance need to be developed to ensure fairness, accuracy and patient trust in AI-powered healthcare solutions (46).

## 2. Augmented reality in cancer detection and management

### Introduction to AR in healthcare. Definition and principles of AR technology

AR technology incorporates information into the real-world situations of the users. It is a set of techniques that combine real and virtual image elements in real-time (50). There are numerous definitions for AR; however, Azuma (51) defined AR as any system that combines real and virtual, one that is interactive in real-time and that is registered in three dimensions. AR has been studied for several years, as this technology continues to evolve and may play a critical role in complex medical interventions. AR, virtual reality (VR) or mixed reality technology falls under the umbrella of what is termed extended reality (XR) (52). AR is an enhanced, interactive digital version of the real-world, which includes visual elements, sound and other sensory stimuli via holographic technology (53). AR differs from VR, in that the user utilizing AR is always exposed to their own reality in real-time (54). The essential components of AR technology can be described as requiring physical-world modeling, display with augmentation and perceptual rendering, interaction and evaluation, such as functional testing, validation and ethics (55).

*History and evolution of AR applications in medicine*. AR first appeared in the 1950s, but was not defined until much later (55,56). In 1962, Heilig built a prototype, which he described in 1955 as ‘The Cinema of the Future’ and in 1968, Ivan Sutherland became the first person to create an AR system using an optical see-through head-mounted display (55). There has been an increased number of publications in recent years as a result of the COVID-19 pandemic in 2020(57). During the COVID-19 pandemic, as individuals were confined to their homes, there was an increase in the practice of telemedicine and performing teaching tasks by creating simulation models and virtual environments (57). Currently, in the healthcare sector, the AR user will be experiencing a superimposition of VR onto clinical images of a real patient in real-time (52). This will allow for the visualization of internal structures through overlying tissues, and provide a virtual transparency vision of the human anatomy (52). This can be particularly important in complex surgical interventions, where the surgeon can benefit from AR. Hidden organs inside a body can be observed by surgeons, and the perception of an interventional procedure can be improved (58). Telemedicine platforms with remote capabilities and AR interfaces are being investigated in several medical and surgical areas (59). Several advantages have been reported when using AR eyewear (60). These include images that can be tracked and the recording of the steps that can be available in the event of an interruption, and the decrease in mental burden of surgeons during a surgical procedure when the surgeon is verifying all the necessary steps were completed (60).

### AR in visualizing tumors and metastases. AR for real-time visualization of tumors in imaging (e.g., 3D reconstructions)

A previous study described the use of the region of interest (ROI), which is a part of the image used for further measurements, such as breast tumors by setting tumor boundaries and determining the size (52). Head and neck cancer specimens bring a challenging task in determining orientation and identification as a result of their 3D structure and the involvement of various tissue structures including mucosa, nerves, blood vessels, muscle, cartilage, bone and glandular structures (61). The pathologist usually communicates the margin status results back to the surgeon without any visual aid, and further re-section is performed prior to reconstruction (61). In this aspect, surgeons and pathologists see the need for new technology to enhance the margin relocation and AR has been studied in various medical specialties such as the use of 3D holographic reconstruction of preoperative imaging studies for intraoperative surgical navigation (61). When diagnosing breast cancer, the workflow includes a series of images and various imaging modalities; in the majority of cases, at an early stage, the tumor is not palpable (62). Therefore, localization techniques are required, including wire-guided, carbon tattooing, radioactive seed localization, radio-occult lesion localization and magnetic seeds, which are considered as standard (62). However, none of these techniques have been proven to be superior in reducing margins (63). Another example is brain cancer diagnosis. It is a challenging task for radiologists to accurately identify suspicious areas in brain MRIs, particularly when differentiating between brain tissue and tumors (64). The main segmentation of tumors is time consuming, and its observer-dependent (64). In a recent study, it was reported the use of AR led to a 98.61% accuracy in brain tumor segmentation (64). That study included the evaluation of data from 496 MRI images, which were used in a multi-platform Unity3D graphic engine, powered by various operating systems (64).

*Enhancing accuracy during surgeries with AR overlays*. The field of neurosurgery has been at the forefront of the surgical use of AR, mainly due to the high reliance on diagnostic imaging techniques, such as MRI and CT (65). A recent study evaluated the utilization of an optically tracked AR display in laparoscopic liver surgery during a simulated surgical task (66). That study comprised of 24 participants, in which half were surgeons and the other half were engineers (66). They tested their performance under three different image guidance conditions, including conventional surgery without guidance, a display with the pre-operative 3D model aligned to the laparoscopic view on a black screen, and a conventional AR overlay (66). Almost all participants preferred the single screen AR overlay, but believe that registration accuracy should be improved as well, as to include the depth perception in the display (66). In the systematic review performed by Pérez-Pachón *et al* (67), the existing tracking and registration methods that can be used by healthcare professionals and researchers in their fields were evaluated (67). The combination of holographic headsets, computer tracking libraries and/or SDKs and game engines have become more available to healthcare professionals and researchers (67). Currently, the research on AR-based image overlay surgery lacks the sufficient level of registration accuracy for their use in clinical practice (67).

*AR in improving biopsy precision and targeting*. In addition to AR being studied for surgical interventions, there are studies about its use in performing biopsies. Sparwasser *et al* (68) performed a retrospective study of 4 patients who obtained an augmented reality smart glasses-assisted targeted biopsy (SMART-TB) of the prostate. Their results reported the more likely detection of prostate cancer than the 12-core systemic biopsy and existing biopsy methods such as MRI-guided targeted biopsy (46.42 vs. 27.1%) (68). Furthermore, the SMART-TB required less biopsy cores than systematic biopsy (n=28 vs. 48) (68). Bettati *et al* (69), used a phantom study to quantify the results after testing the accuracy of AR-guided biopsy system and procedure for soft tissue and lung lesions. An average error was found of 0.75 cm from the center of the lesion when AR guidance was used, compared to an error of 1.52 cm from the center of the lesion during unguided biopsy for soft tissue lesions (69). In addition, upon testing the system on lung lesions, an average error of 0.62 cm from the center of the tumor while using AR guidance vs. a 1.12 cm error, while relying on unguided biopsies (69). They concluded that the AR-guided system can improve the accuracy and precision in biopsy acquisition (69). Limited field view, high hologram drift after significant user motion, and the requirement of fiduciary markers were the reported limitations (69). In a recent study, an AR-guided biopsy system was built by utilizing high-speed (IR) and it was evaluated for biopsy acquisition (70). This system consists of HoloLens2, the Unity game design software, and an optical motion capture camera system (70). In that study, the authors developed a highly accurate motion tracking system by using infrared trackers on the biopsy tool and resulted in significantly improved accuracy (2.94±1.04 mm mean targeting error) (70).

### AR for surgical planning and navigation. AR-assisted surgery in metastatic cancers (e.g., liver, brain and lung metastases)

Recently, there have been studies testing the accuracy and precision of AR technologies in surgery. Benbelkacem *et al* (71) discussed the creation of a new tool for the diagnosis of lung tumors by thermal touch feedback using AR visualization and thermal technology. The goal was to assist in identifying and locating tumors, and measuring tumor size and temperature of the tumor surface (71). The medical team who tested this technology reported that the thermal feedback could be useful to locate and better understand the tumor (71). In another prospective pilot study, the authors assessed the potential of AR technology to help locate and surgically resect missing colorectal cancer liver metastases (CRLM) (72). The AR model was superimposed to the operative field using an Exoscope, and all the missing metastases were identified under AR and successfully resected under AR guidance (72). There were 4 patients who had a follow-up period of 24, 22 and 6 months (72). There was no mortality during this period and patients nos. 1 and number 3 were disease-free with patient no. 2 presented with a single CRLM recurrence outside of the sites resected 7 months following the surgery (72). In another study on 10 patients who had intramural spinal tumors, AR was provided by head-up displays of operating microscopes (73). The items visualized by AR were segmented in pre-operative imaging data and in all cases, surgery assisted by AR provided visualization of the tumor outline and other relevant surrounding structures (73). The overall AR registration error was 0.72±0.24 mm (mean ± standard deviation), a close matching of visible tumor outline and AR visualization was observed for all cases.

*Enhanced navigation during complex procedures using AR systems*. Some studies have reported how AR systems can improve in accuracy and visualization of the body anatomy in complex procedures, mainly surgical. Neurosurgeons face challenges constantly in the operating room by dealing with fragile blood vessels, critical neural structures (73), and they depend on imaging in the preoperative planning. For example, in neurooncology, the use of VR/AR for neuronavigation has been shown to improve the extent of resection and overall survival (74). In another study, combining VR and AR with intraoperative MRI and functional imaging enhanced the extent of tumor resection, while preserving neural function in patients with gliomas (75). Gliomas are the most common intraaxial tumors, and it is challenging to distinguish them from surrounding brain tissue (75). Intraoperative brain imaging validates intraoperative ultrasound through AR (74). A previous study performed 20 head and neck cancer re-resections to determine the accuracy and feasibility of AR surgery guidance (61). The head and neck resection specimen were 3D-scanned and exported to the HoloLens AR environment (61). Complex oral cavity composite resections (maxillectomy and mandibulectomy) had a higher mean relocation error than all the other specimens (10.7 vs. 2.8 mm; P=0.0003) (61). That study supported that 3D specimen holograms can improve the relocation accuracy (61). In another laboratory-based experiment, a patient-specific manikin was used including pre-operative MRI images to simulate a craniotomy procedure (76). The VOSTARS head-mounted display was used to plan the craniotomy (76). That study suggested that AR assisted in simulating convexity en plaque meningioma resection and cranioplasty (76). Neurosurgery has been in the forefront of AR technology; however, more studies are needed to support AR potential use.

### AR in patient education and decision-making. AR tools to improve patient understanding of their diagnosis and treatment options

It is imperative for the patient undergoing treatment and/or surgery to understand the implication of the treatment and options available to make informed decisions that are in their best interest. The AR user can display 3D models of the anatomy of the patient for patient and family education. The study by Wake *et al* (77) evaluated how 3D models of renal and prostate cancer can impact patient education. Their study consisted of 200 patients who completed a survey after reviewing their individual cases with their surgeons using imaging only (77). A total of 127 patients completed the 5-point Likert scale survey regarding the understanding of disease and surgical procedure twice, once with imaging and again after reviewing imaging plus a 3D model (77). Patients reported greater understanding using 3D printed models vs. images (range, 4.60-4.78/5 vs. 4.06-4.49/5, P<0.05) (77). In another study by Wang *et al* (78), a total of 75 patients participated in an AR simulation where they viewed 3D breast tangents and intensity modulated radiotherapy lung plans using a portable iOS and android device. This patient education application allowed patients to view a treatment simulation (78). A total of 35 patients expressed anxiety about radiotherapy beforehand; however, 21 (60%) indicated a decrease in anxiety after the AR session (78). However, these studies had limitations, including the fact that patient questionnaires with imaging were conducted first, followed by the introduction of 3D models, which may have enhanced patient understanding due to repetition rather than the use of the 3D models themselves. Additionally, the second study lacked a control group for comparison (77,78). In the study by Tait *et al* (79), 91 parent/child dyads (children from ages 7-13 years) were randomized to receive the basic information about clinical research using printed storybook (control) or the same storybook enhanced using a video see-through AR iPad program with interactive quizzes (79). Children were interviewed pre- and post-test, and both parents and children completed surveys to measure their understanding (79). The findings demonstrated that parents of children in the AR group rated the information as having higher quality and clarity compared to the control group (79). Additionally, 91.7% of children in the AR group found the interactive quizzes helpful, and both parents and children found the AR program very easy to use (85.0 and 71.2%, respectively) (79).

*Improving patient communication through immersive visualizations*. Enhancing communication between patient and staff participating in their care provides many benefits including clearing out any questions or concerns that the patient may have, mitigates anxiety of certain treatments and surgical procedures, and increases patient compliance. The study performed by Brown *et al* (80), evaluated the effectiveness of using a mobile phone-based nutrition tool, ServARpreg, in carbohydrate and standard serve size education in pregnant women for blood glucose regulation. During the evaluation survey, 80% strongly agreed/agreed that ServARpreg made them more aware of their portion size and 72.5% found it user-friendly (80). In a radiation oncology study by Wang *et al* (78), the majority of the patients (95%) indicated that AR viewing provided them with a greater understanding of how radiotherapy will be used as their treatment. Their system allows the user to experience a 3D animation, which was loaded onto their smartphone for repeat viewing later to reinforce the learning (78). In another single-site study of 100 randomized patients diagnosed with kidney masses or stones, a physician used AR software on a tablet for patient education, presenting 3D images of kidney cancer and stones (81). These were compared to the standard of care (81). They concluded that there was no significant difference pre- to post-visit improvement in self-reported understanding between arms; however they reported that the patient satisfaction was higher in the AR group (81).

### Limitations and challenges of AR in cancer management. Technological barriers (e.g., hardware, software and cost)

AR systems often include a navigation system, which poses challenges, such as patient and respiratory motion, sterile registration, soft tissue and organ deformation and needle bending (82) in interventional radiology for example. These navigation systems are costly, and their use is not reimbursable for interventional radiology procedures in the USA (82). Sparwasser *et al* (68) reported how SMART TB (augmented reality smart glasses) can assist in targeted biopsy of the prostate. Some benefits reported included that time- and cost-effectiveness, however detection rates using this technology were highly dependent on the surgeon performing the procedure (68). Other studies have reported limitations in using AR Head-Mounted Displays (HMDs), including perceptual conflicts between the real and virtual world images, the small field of view, the sub-optimal ergonomics and calibration issues (59). In another study, the authors created custom software that automates the process of taking standard DICOM-RT data from their radiotherapy treatment planning system and converting it into patient-specific AR animations into other hardware platforms making it cost-efficient (78). In addition, cyber security is imperative for the digitization of data in healthcare including safe data storage, data transfer, confidentiality, unauthorized access and the use of patent data (82). Roethe *et al* (83) assessed the overall surgical use of AR in 55 intracranial lesions, operated either with AR-navigated microscope (n=39) or conventional neuronavigation (n=16). They demonstrated that the majority of participants preferred a peripheral display of information over AR visualization in the focus level as a result of visual occlusion and reported distraction effect (83). In addition, an AR device can increase neck discomfort from the weight, for example a headset. Few evaluations of cost-effectiveness have been performed (84).

*User acceptance (e.g., by surgeons, oncologists and patients)*. AR has been used within the field of radiology as an educational tool for medical students and residents to understand human anatomy (82). In addition, there are existing interactive platforms that allow remote consultants to project live annotations into the AR display of another operator, providing expert assistance (82). In the study by Csaxner *et al* (85), a user study was conducted assessing a novel AR system for visualization of medical image data saved with the head or face of patients with head and neck carcinoma, and revealed above average usability with a SUS score of 74.8, with a score >70, implying acceptance by the target audience. The users in that study reported the system easy to control (85). Wake *et al* (77) reported a greater patient understanding of their disease and surgical procedure when it was discussed using 3D printed models and AR. Surgeons require assistance in observing hidden organs and their complex surroundings to accurately perform a procedure. The acceptance of AR in clinical applications is limited due to its technical and clinical challenges (85). Tait *et al* (79) described the importance in providing information to research participants and patients in an easy-to-read manner, and their study resulted in AR being the preferred method of information delivery amongst participants. However, further studies are warranted to continue to evaluate the limitations that AR carries.

## 3. Synergy between AI and AR in metastatic cancer management

### Integrating AI with AR technologies. How AI-driven algorithms can enhance AR visualization (e.g., dynamic tumor tracking during surgery)

The concept of AI may be summarized as the combination of vast amounts of data, powerful computers, and clever algorithms to create models that solve certain problems and mimic human thought processes (86). Using computer graphics and visualization technologies, AR creates overlaying digital content onto the actual environment of the user, creating an interactive and enriched experience that does not replace reality. This enables AI to analyze and evaluate intraoperative pictures in real-time, plan the surgical path and simulate the surgical procedure. Reverse reality technology or non-realistic rendering are the primary methods used by AR systems to render and show the pre-existing virtual representation. A synergistic effect known as ‘1+1 >2’ can be achieved by the development and integration of AI and AR in surgical devices to differing degrees. This includes enabling intelligent matching and precise positioning and 3D dynamic observation, displaying the depth and angle of the surgical path, and monitoring the surgical environment and procedure (87). AI is therefore very useful in all areas of healthcare. AI and AR technologies have recently been widely used in a variety of procedures, including in cardiovascular (88), gastrointestinal (89) and policing (88-90). The development of AR successfully addresses the difficulty of how to comfortably apply AI findings to the actual world by superimposing AI-based information into a sample's present view in real-time, allowing for the seamless integration of AI with daily workflows.

*Combining AI and AR for better decision-making support in real-time*. A number of aspects of contemporary life have undergone a rapid transformation in recent years due to ever-increasing technological innovation. When combined, AR and AI technology can help police officers who must make tough choices under duress by providing vital information that is immediately relevant to the current circumstance (90).

In order to achieve technology-assisted policing in ways that were previously only possible in science fiction, DARLENE, an EU-funded Research and Innovation Action, combines the benefits of both AR and AI (91).

The potential applications of XR technology for illness detection and therapy require further investigation. According to previous studies, XR technology, which includes VR and AR, is just as successful in enhancing patient care and learning outcomes as conventional teaching techniques (92,93). While AR superimposes these insights onto the actual physical nvironment, AI is capable of processing enormous volumes of data and producing actionable insights. In addition to healthcare, AR overlays driven by AI may direct personnel in manufacturing. AI-powered traffic analysis aids in urban planning. Additionally, it supports retail and e-commerce as well as emergency response to disaster areas (92,93).

### Applications in multidisciplinary care. Collaborative benefits in oncology: AI/AR for multidisciplinary teams (surgeons, oncologists and radiologists)

To enhance diagnosis, prognosis and treatment results, the new area of radiogenomics integrates molecular pathology with medical imaging. The fact that there is a substantial amount of ‘untapped’ therapeutically useful data in medical imaging has recently come to light (94). Additionally, the advancement of radiogenomics may be essential to IR and its use in the treatment of patients with cancer, including those with lung cancer, colorectal cancer with liver metastases, renal cell carcinoma (RCC) and hepatocellular carcinoma (HCC). Studies on RCC have demonstrated links between CT imaging features and tumor mutations and, consequently, clinical outcome, and radiogenomic research has shown promise in linking HCC gene patterns with aggressive CT imaging features, such as infiltration or microvascular invasion. Complex medical data and images are analyzed by AI systems. This method enhances the prognosis and characterization of tumors.

Radionics has been utilized to link imaging characteristics with the presence of RAS mutations in colorectal cancer (95). In addition, the creation of the AR microscope (ARM) incorporates AI to seamlessly integrate into the microscopy process by superimposing diagnostic data in real-time over the sample's present image. This invention has proven useful in recognizing prostate cancer and detecting lymph node metastases in breast cancer (96). The combination of AI and AR encourages collaboration between radiologists, pathologists and oncologists. This guarantees the thorough analysis of patient data, resulting in treatment programs that are better informed and more successful. For instance, research has shown a low participation in head and neck cancer care, despite the acknowledged potential of AI to improve diagnostic accuracy and treatment choices (97). This underscores the need for wider use and further studies to examine its acceptability and advantages. Data quality, interoperability, standardization, and ethical issues are some of the difficulties in incorporating AI and AR into clinical practice. Collaboration between researchers, physicians, data scientists, and industry stakeholders is necessary to overcome these challenges.

### Future prospects and innovations. Advancements in AR and AI integration

Enhancing user experiences and increasing application options are two major benefits of combining AI with AR. The fusion takes a multidisciplinary approach in a medical environment. Urology is one of the many medical specialties that stand to benefit greatly from the adoption of modern medical technologies (98), particularly medical enhanced AR (MER) headsets. The introduction of AR headsets, such the Oculus and Apple Vision Pro models, has the potential to completely transform a wide range of medical applications when incorporated into clinical settings. Laparoscopic urology operations will be made simpler and more accurate by the ability of MER to superimpose CT images on the operating field. With the ability to seamlessly overlay 3D MRI images with the patient's anatomy to direct the surgeon during percutaneous kidney puncture, MER technology also exhibits potential in endourology (99).

It may be possible to more precisely prognosticate and forecast a the reaction of a patient to a given treatment using radiogenomic-based triage techniques, resulting in a more specialized and targeted treatment strategy. These enhance the clinical function of interventional radiologists. Since the development of AI in post-procedural follow-up continues to enable more the specialized and customized treatment of patients with cancer, interventional oncology stands to gain a great deal (100,101).

In addition to providing neurosurgeons with rapid and efficient tools for pre-, post- and intraoperative care, AI can improve the accuracy of diagnosis and treatment in the field of neurosurgery. AI helps neurosurgeons identify minor anomalies and deformities from neuroradiological pictures and clinical data that even highly skilled human eyes can miss (102).

*Potential future applications of AI and AR in personalized cancer care*. AR and AI have the potential to completely transform individualized cancer treatment. AI is being used in a large-scale experiment by the UK National Health Service (NHS) to enhance breast cancer detection, which may lessen the workload of radiologists and speed up diagnosis. By examining variables including genetics and lifestyle, AI models can forecast a the risk of an individual to develop cancer, allowing for more individualized screening methods. AI is capable of analyzing patient data to customize treatment regimens, enhancing results and minimizing adverse effects (103). By using large datasets and customized treatments, the promoted combination of AI and AR speeds up drug discovery (104). Applications powered by AI can speed up AR and help patients manage their symptoms and post-treatment lifestyles. Surgeons benefit from the enhanced accuracy of AR and 3D tumor visualization. Medical practitioners may rehearse intricate cancer procedures using the realistic simulations of AR. These developments in AI and AR have the potential to revolutionize customized cancer care by enabling improved patient experiences, more effective therapies and earlier detection (105,106).

## 4. Ethical, social and regulatory considerations

### Ethical implications of AI in cancer care. Impact of AI on patient autonomy, trust and data privacy

There are critical ethical implications as regards the use of AR in cancer care, including legal and privacy issues. As in a number of invasive procedures, informed consent from the patient is of utmost importance to provide the patient with autonomy, and to protect the confidential information of the patient. It is the responsibility of the user to ensure that patients being exposed to these technologies understand the purpose, duration, complications and expectations of their use. An additional key point is that the technological ability and capacity of patients to engage with these advancements can lead to inequality in the delivery of healthcare (87). A recent US study found that general support for AI was higher amongst those who were healthy, male, educated, or experienced in technology (107). When using this technology, there is a concern when dealing with patient confidentiality when handling the health records of patients. Cyber security is an essential consideration of digitization of data (87). In addition, implementing AI may narrow the options that are offered to patients as regards treatment by considering research, personal health and non-health data, leaving the patients with a narrower range of options (107). Lastly, developing trust between patient and healthcare professionals as a one-to-one relationship instead of using only AI, is one of the most critical aspects in healthcare to be able to provide the optimal care for the optimal outcomes. This is particularly important in oncology, where human contact and empathy are critical.

*Bias in AI algorithms and its effects on treatment outcomes*. Bias in AI algorithms is one of the known problems in providing care to patients. Therefore, AI systems used for the evaluation of breast cancer care need to pay close attention to the effects in different cohorts of women (107). According to Carter *et al* (107), the first task for AI systems should be the current human task of reading large volumes of images, such as digital mammograms or histopathological slides. In this circumstance, AI will still depend on human performance and will provide an opportunity to augment rather than replace human function (107). There are ethical concerns related to the biases that may exist in the algorithms used by AR systems (108). These biases can lead to unequal treatment outcomes, mainly if the technology is unable to account for diverse patient populations with varying skin tones, facial structures and characteristics (108). The immerse nature of AR and VR may lead to patients expecting certain outcomes that are not feasible and can create dissatisfaction in patients (108). Team developing algorithms should be explicitly aware of the specificities of the health system for which they are creating these algorithms considering the needs of different groups (109) and tailor healthcare to those groups for better health outcomes and equality. Recommendations are being made by implementing clinical expertise to propose relevant errors for the context in which the algorithm is being developed (109).

### Regulatory and legal challenges. Regulatory approval processes for AI and AR technologies in healthcare

The FDA regulatory requirement will not apply if the device has certain functions solely intended to transfer, store, convert formats, or display medical device data and results, including medical images, waveforms, signals, or other clinical information (110). The Medical Extended Reality Program in the FDA's Center for Devices and Radiological Health (CDRH) conducts regulatory science research to ensure patient access to safe and effective innovative extended reality-based devices (110). When it comes to FDA review and approval, AI-enabled medical devices have three pathways, including *de novo*, premarket clearance [510(k)] and premarket approval (111). *De novo* pathway is for low-to-moderate-risk medical devices with no predicates, as for example, referenceable devices that are of the same type as the applicant's product but already approved by the FDA. Premarket clearance [510(k)] pathway is for moderate-to high-risk medical devices with market predicates and equivalent safety and effectiveness. Premarket approval pathway is for high-risk medical devices, which requires that the clinical investigations in the submission address various factors (111). The FDA regulates software that performs a medical function independently of the hardware on which it operates as ‘software as a medical device’ (SaMD) (111). The FDA then further distinguishes off-the-shelf (OTS) software that may be used within a medical device and the supplier of the OTS software is not regulated by the FDA (111). However, the medical device manufacturer bears the responsibility for the continued safe and effective performance of the medical device (108). The manufacturer has to comply with the Health Insurance Portability and Accountability Act (HIPAA) and the Health Information Technology for Economic and Clinical Health (HITECH) Act where these devices receive, store, maintain and transmit protected health information (111).

*Standardization and safety concerns*. The US FDA have identified 10 guiding principles that can inform the development of Good Machine Learning Practice (GMLP) (110). The goal of these guidelines is to ensure the safety, effectiveness and high-quality of medical devices that use artificial intelligence and machine learning (AI/ML) (110). The 10 principles include the multi-disciplinary expertise is leveraged throughout the total product life cycle; good software engineering and security practices are implemented; clinical study participants and data sets are representative of the intended patient population; training data sets are independent of test sets; selected reference datasets are based upon best available methods; model design is tailored to the available data and reflects the intended use of the device; focus is placed on the performance of the human-AI team; testing demonstrates device performance during clinically relevant conditions, users are provided clear, essential information; deployed models are monitored for performance and re-training risks are managed (110). AI devices need to also comply with HIPAA in the event that they handle the confidential information of patients, as with other devices approved for patient safety. Manufacturers of AI devices used in healthcare must ensure that the device includes software that has been validated, installed, or authorized by the sponsor (110). The device should also be capable of connecting to the internet and contain any technological characteristics validated, installed, or authorized by the sponsor. In the event that there is any uncertainty about whether the device qualifies as a cyber device or includes these features, the FDA should be contacted (110).

### Equity and accessibility. Access to AI and AR technology in low-resource settings

AR technology can be costly and therefore unavailable to low-income and low-resource community hospitals, clinics and other healthcare settings. These communities are currently falling behind on these AI technologies, regardless of research evaluating their use in healthcare. However, AI can be impactful in various domains and it has been shown that developing countries emphasize using AI in education, healthcare and government (112). In the health domain, X-rays are a standard medical procedure performed in all countries, and AI tools developed to process X-rays and diagnose diseases can be easily transferred to low-income countries (112). Some AI systems are deployed in hardware; for example, they are embedded within the CT scanner, US unit, or mammography station, while others are deployed by using PACS as the delivery platform (113). In addition, AI technologies can be used to promote telemedicine in some low-income countries, which can extend the access of medical services to remote areas (112). In the education sector, education outcomes in these countries are worse, where approximately 258 million children, adolescents and youth were out of school in 2018 as reported by UNESCO (112). Therefore, AI usage can mitigate these challenges by providing other methods of learning, such as distance, online and personalized learning (112). However, implementing AI in education may be challenging due to limited access to technology and the internet in these countries (112). Establishing collaborative networks with advanced economies and international organizations can enhance technology and knowledge transfer to accelerate the learning curve for low-income countries (112).

*Potential for healthcare disparities and the digital divide*. Mollura *et al* (113) introduced RAD-AID, a strategy for AI adoption in resource-poor health institutions that accounts for clinical radiology education, infrastructure implementation and phased AI introduction. Accessibility is critical for AI in resource-poor health institutions. An example of the work of RAD-AID in building radiology infrastructure in these institutions is the RAD-AID Friendship PACS program, which includes a donated on-site server, web-enabled PACS software, and cloud infrastructure as a platform that can deliver and run AI applications (113). RAD-AID has supported mobile radiology outreach and therefore, increased access to conventional radiology services (113). AI-based healthcare models need to be incorporated with data of diverse populations, including underrepresented communities. Tailoring the care to diverse communities is crucial for improved health outcomes. However, there are certain populations that lack access to technology, have insufficient broadband access, with worse access for the disabled and the use of lower performing devices (114). A low income, the female sex, and being of African ethnicity have been shown to be associated with a decreased probability of completing a telehealth visit (114). For example, in Nigeria, rural areas have limited internet connectivity, inadequate infrastructure, socioeconomic constraints and disparities in digital literacy (115). Rural Nigerians face challenges in accessing vital health information, mainly with the emergence of AI technologies causing disparities in AI health information access (115).

Recent advances in AI have notably impacted colorectal cancer diagnosis, particularly through the application of DL techniques. DL algorithms have demonstrated significant promise in enhancing colorectal cancer histopathology classification by automating the detection and characterization of cancerous tissues with high accuracy and efficiency. These algorithms can analyze large volumes of histopathological images, identifying subtle morphological features that may be overlooked by human observers, thereby improving diagnostic precision and aiding pathologists in clinical decision-making. The studies by Bousis *et al* (116) and Chlorogiannis *et al* (117) emphasize the growing potential of these approaches to be integrated into routine clinical workflows, potentially reducing diagnostic variability and accelerating patient management.

In addition to DL, the internet of things (IoT) is emerging as a transformative tool in cancer detection and management. IoT-enabled devices facilitate the continuous, real-time monitoring of patient health parameters and environmental factors, thereby supporting early detection of cancer-related changes and personalized treatment plans. The integration of IoT in oncology allows for seamless data collection and remote patient monitoring, which can enhance adherence to therapy, enable timely interventions, and improve overall outcomes. Recent reviews underscore the role of IoT in creating interconnected healthcare ecosystems that empower clinicians with actionable insight, while enhancing patient engagement and quality of care (118). The convergence of deep learning and IoT technologies represents a promising frontier in the management of colorectal cancer, with the potential to revolutionize diagnostic accuracy and patient-centric care.

## 5. Addressing technical implementation, limitations, ethical issues and future directions

To enhance the practical impact of AI and AR technologies, future studies are required to elaborate on implementation details, such as AI model architectures, data preprocessing pipelines and AR system integration. For instance, CNNs and transformer-based architectures are increasingly used for histopathology image analysis in colorectal cancer, enabling early diagnosis through automated pattern recognition. Similarly, AR-assisted surgical navigation systems are improving intraoperative precision by guiding tumor resections with real-time 3D overlays.

Despite these advances, significant technical and ethical limitations persist. Key challenges include data heterogeneity, algorithmic bias and the complexity of integrating these tools into routine clinical workflows. Ethical issues, such as patient data privacy, informed consent and accountability remain at the forefront of debate.

Real-life incidents underscore these concerns. For example, a 2019 data breach at the University of California, San Francisco (UCSF) compromised thousands of patient imaging records, highlighting vulnerabilities in healthcare data storage and emphasizing the need for secure encryption and controlled data access; this was previously discussed in a previous study (119). Similarly, an AI dermatology algorithm developed by a major tech company demonstrated reduced accuracy for darker skin tones, revealing how underrepresentation in training datasets can perpetuate diagnostic inequities and harm minority patient groups (120). These examples illustrate the dual risks of data insecurity and algorithmic bias that can erode trust in AI-driven healthcare systems.

Proposed solutions include implementing standardized data governance frameworks that define ownership, access control and accountability for shared medical data. Continuous model validation and retraining using diverse, representative datasets can mitigate bias and improve generalizability. Additionally, establishing regulatory and ethical oversight, clarifying responsibility among developers, clinicians and institutions, will be crucial for ensuring transparency and patient safety in AI-assisted decision-making.

Future research is warranted to prioritize the development of interpretable and robust AI models that can explain their predictions, as well as interoperable AI-AR platforms designed for seamless clinical integration. Multi-institutional collaborations leveraging large, diverse datasets will enhance both fairness and reliability. Emphasizing ethical AI design, stakeholder engagement, and rigorous clinical validation will be key to translating these technologies safely and equitably into medical practice.

## 6. Conclusion

Metastatic cancer remains a global health challenge, characterized by late detection, therapeutic resistance and heterogenous disease progression. The integration of AI and AR provides a potential solution to address these issues. AI enhances diagnostic precision by analyzing complex datasets, which enables the early identification of micrometastases and personalized treatment strategies. Concurrently, AR improves intraoperative accuracy through real time 3D tumor visualization, dynamic surgical navigation, and impressive patient education, fostering informed decision-making and reducing procedural risks. The synergy between AI-driven predictive analytics and AR-enhanced visualization supports multidisciplinary collaboration, optimizing therapeutic planning and executing across oncology, radiology and surgery.

However, challenges such as algorithmic bias, data privacy risks and regulatory hurdles persist, alongside disparities in resource limited settings. Future advancements must prioritize ethical AI frameworks, standardized protocols, worldwide partnerships to ensure equitable implementations. Innovations such as AI-driven tumor tracking and AR-enhanced telemedicine could further refine precision oncology. By integrating these technologies with patient centered care, there is potential to improve survival outcomes and reduce global burden of metastatic cancer.

## Data Availability

Not applicable.
